# Seroprevalence of cytomegalovirus and its coinfection with Epstein-Barr virus in adult residents from Manaus: a population-based study

**DOI:** 10.1590/0037-8682-0363-2019

**Published:** 2020-01-27

**Authors:** Gustavo Magno Baldin Tiguman, Laura Beatrice Poll, Carlos Eduardo de Castro Alves, Gemilson Soares Pontes, Marcus Tolentino Silva, Tais Freire Galvao

**Affiliations:** 1 Universidade Estadual de Campinas, Faculdade de Ciências Farmacêuticas, Campinas, SP, Brasil.; 2 Instituto Nacional de Pesquisas da Amazônia, Manaus, AM, Brasil.; 3 Universidade de Sorocaba, Programa de Pós-Graduação em Ciências Farmacêuticas, Sorocaba, SP, Brasil.

**Keywords:** Cytomegalovirus, Seroprevalence, Epstein**-**Barr virus, Coinfection, Amazon

## Abstract

**INTRODUCTION:**

This study assessed the seroprevalence of cytomegalovirus, associated factors, and Epstein**-**Barr virus coinfection among adult residents of Manaus.

**METHODS:**

Using a cross-sectional study design, we collected blood samples from 136 individuals in a household survey in 2016. Prevalence ratios were calculated using Poisson regression.

**RESULTS:**

Cytomegalovirus and Epstein**-**Barr virus seroprevalences were 67.6% (95% CI: 9.7-75.6%) and 97.8% (95% CI: 95.3-100.0%), respectively. Coinfection was observed in 66.2% (95% CI: 58.1-74.2%) of participants. Bivariate analysis showed no statistical association.

**CONCLUSIONS:**

Seroprevalences were high among participants and approximately 7 out of 10 individuals had cytomegalovirus and Epstein**-**Barr virus coinfection.

Cytomegalovirus seroprevalence in adults ranges between 60% in developed countries and 100% in developing countries^1^. Infections are more frequent in low socioeconomic status groups, due to lower levels of education, and poor hygiene and sanitation conditions^1^. High socioeconomic levels have been associated with lower seroprevalence and higher susceptibility to new infections, whereas individuals from low-income groups have higher seroprevalence and less susceptibility to new infections due to the higher frequency of cytomegalovirus antibodies. After the first infection, the virus may remain latent and can later lead to new infections, especially in immunocompromised individuals^2^.

Cases of coinfection are common as the prevalence of cytomegalovirus is high; a common pathogen found in these cases is the Epstein**-**Barr virus, which also belongs to the *Herpesviridae* family. The virus is the etiological agent of infectious mononucleosis, transmitted mainly by oral secretions^3^. It is found in more than 90% of the global population, with its prevalence varying according to the development status of countries. Infections occur more frequently in newborns and they are asymptomatic. However, in industrialized countries, primary infection usually occurs in adolescence or adulthood, leading to infectious mononucleosis^3^.

The aim of this study was to assess the seroprevalence of cytomegalovirus, associated factors, and coinfection with Epstein**-**Barr virus in adult residents of Manaus, Amazonas. To the best of our knowledge, this is the first study to investigate the prevalence of these infections in a population from this region.

This was a cross-sectional seroepidemiological study conducted from September to December 2016, with adults that had previously participated in a major population-based survey carried out in Manaus Metropolitan Region^4^. The previous survey included 4,001 adults (≥18 years), selected using a probabilistic three-step complex sampling design.

Participants from the original survey were invited by telephone to participate in the serologic study. They were contacted at least twice to schedule a household visit, according to their availability. All participants that agreed to take part in this serologic study provided written consent. We attempted to include all individuals who participated in the population-basedw survey from 2015. Thus, sample size estimation was not calculated for our study. 

The primary outcome was defined as the prevalence of cytomegalovirus infection, assessed through the detection of anti-cytomegalovirus immunoglobulin G (IgG) in the plasma samples of the participants. Active cytomegalovirus infection was also investigated by testing the samples for anti-cytomegalovirus immunoglobulin M (IgM). The secondary outcome was the prevalence of Epstein**-**Barr virus, assessed by the detection of virus-specific IgG. 

The independent variables investigated were: sex (female, male), age group (18-34, 35-49, and ≥ 50 years), ethnicity (white, non-white), number of household members (1-3, 4-6, 7-25), educational level (higher education or above, high school, elementary school, less than elementary school), economic status (A/B, C, or D/E, where A refers to the wealthiest and E to the poorest), health insurance coverage (yes, no), chronic diseases (yes or no for hypertension, diabetes, high cholesterol, cardiovascular disease, stroke, asthma, rheumatoid arthritis, chronic back pain, depression, mental illness, respiratory disease, cancer, chronic renal failure, and others), and diagnosis of malaria (yes, no) and dengue (yes, no) in the previous 12 months.

Peripheral blood samples from 136 participants were collected in ethylenediaminetetraacetic acid (EDTA)-containing tubes (15% potassium EDTA with 0.34 mmol/L aprotinin). After blood fractionation, plasma samples were stored at - 80 °C until the analysis was performed.

The plasma samples were analyzed by an enzyme-linked immunosorbent assay to detect anti-cytomegalovirus IgM or IgG. The tests were performed according to the manufacturer’s instructions (Serion ELISA classic, SerionGmbH, Germany). Optical densities of the test samples were measured using a microplate reader at a wavelength of 450 nm and positivity for anti-cytomegalovirus IgM or IgG was estimated according to the cut-off value provided by the manufacturer’s protocol. The cut-off ranges were estimated by multiplying the mean value of optical densities (OD) of the positive controls with the numerical data from the quality control certificate (OD=0.600 x positive control mean for upper cut-off; OD=0.350 x positive control mean for lower cut-off). For example, when the mean absorbance value of positive controls was 0.850, the cut-off values would range between 0.290-0.510. All tests were performed at the Laboratory of Immunology and Virology of the National Institute of Amazonian Research (INPA), Manaus, Brazil. 

Prevalences of cytomegalovirus and Epstein**-**Barr virus, with 95% confidence intervals (95% CI), were calculated along with other descriptive statistics. Prevalence ratios (PR) were calculated using Poisson regression with robust variance to assess if any variable was associated with cytomegalovirus seropositivity in the bivariate analysis. For the variables that showed statistical significance at the level of p<0.20, a multivariate analysis was planned. Associations were considered statistically significant if the p-value was <0.05. Data analyses were performed using Stata V.14.2 (Stata). 

This project was approved by the Research Ethics Committee of the Federal University of Amazonas (Opinion number: 1,541,710, on 12 May 2016; *Certificado de Apresentação para Apreciação Ética* - CAAE: 42203615.4.0000.5020).

Of the 4,001 individuals who were interviewed in the population-based survey, 136 agreed to participate in the seroepidemiological study, of which 92 were positive for cytomegalovirus (67.6%; 95% CI: 9.7%-75.6%) and 133 were positive for Epstein**-**Barr virus (97.8%; 95% CI: 95.3%-100.0%). Coinfection of cytomegalovirus and Epstein**-**Barr virus was found in 66.2% (95% CI: 58.1-74.2%) of participants.

As described in Table 1, most individuals in the study were females (n=80; 58.8%), aged 18-49 years (n=98; 72.1%), non-white (n=101; 74.3%), lived in a household with 1-6 residents (n=120; 88.2%), had completed at least high school (n=78; 57.4%), belonged to the lower economic groups (C, D/E; n=103; 75.7%), had no health insurance (n=124; 91.2%), had no diagnosis of malaria in the previous 12 months (n=132; 97.1%), and had no diagnosis of dengue (n=129; 94.9%). Chronic diseases were reported by 68.4% (n=93). These included chronic back pain (n=55; 40.4%), hypertension (n=38; 27.9%), diabetes (n=11; 8.1%), high cholesterol (n=32; 23.5%), rheumatoid arthritis (n=29; 21.3%), asthma (n=15; 11.0%), depression (n=12; 8.8%), cardiovascular disease (n=11; 8.1%), mental illness (n=8; 5.9%), stroke (n=5; 3.7%), respiratory disease (n=3; 2.2%), cancer (n=2; 1.4%), chronic renal diseases (n=2; 1.4%), and other chronic diseases (n=9; 6.6%).

**TABLE 1:** Frequency of cytomegalovirus infections (CMV) and prevalence ratios (PR) with 95% confidence intervals (95% CI) for the socio-demographic and clinical characteristics of adults living in Manaus, 2016.

Variables	Total (n=136)		CMV (n=92)		PR (95% CI)	p-value
	n	%	n	%		
**Sex**						0.654
Female	80	58.8	52	65.0	1.00	
Male	56	41.2	40	71.4	1.10 (0.73-1.66)	
**Age group (years)**						0.945
18-34	47	34.6	33	70.2	1.00	
35-49	51	37.5	33	64.7	0.92 (0.57-1.49)	
≥ 50	38	27.9	26	68.4	0.97 (0.58-1.63)	
**Ethnicity**						0.382
Nonwhite	101	74.3	72	71.3	1.00	
White	35	25.7	20	57.2	0.80 (0.49-1.32)	
**Household members**						0.973
1-3	43	31.6	30	69.8	1.00	
4-6	77	56.6	51	66.2	0.95 (0.60-1.49)	
7-25	16	11.8	11	68.8	0.99 (0.49-1.97)	
**Educational level**						0.718
Higher education or above	11	8.1	8	72.7	1.00	
High school	67	49.3	44	65.7	0.74 (0.37-1.47)	
Elementary school	19	14.0	13	68.3	0.81 (0.36-1.82)	
Less than elementary school	39	28.7	27	69.2	0.65 (0.31-1.36)	
**Economic status**						0.993
A/B	33	24.3	22	66.7	1.00	
C	79	58.1	54	68.4	1.03 (0.62-1.68)	
D/E	24	17.7	16	66.7	1.00 (0.53-1.90)	
**Health insurance**						0.746
No	124	91.2	83	66.9	1.00	
Yes	12	8.8	9	75.0	1.12 (0.56-2.23)	
**Chronic diseases**						0.489
No	43	31.6	26	60.5	1.00	
Yes	93	68.4	66	71.0	1.17 (0.75-1.85)	
Malaria*						0.856
No	132	97.1	89	67.4	1.00	
Yes	4	2.9	3	75.0	1.11 (0.35-3.51)	
Dengue*						0.729
No	129	94.9	88	68.2	1.00	
Yes	7	5.2	4	57.1	0.84 (0.31-2.28)	
Epstein-Barr vírus**						0.983
No	3	2.2	2	66.7	1.00	
Yes	133	97.8	90	67.7	1.02 (0.25-4.12)	

*Self-reported, for the previous 12 months. **Epstein**-**Barr virus seropositivity was assessed by testing the participants’ blood samples.

Cytomegalovirus was more frequent among men (71.4%), individuals aged 18-34 years (70.2%), non-white participants (71.3%), those with health insurance (75.0%), individuals with concomitant chronic diseases (71.0%), participants with a diagnosis of malaria in the previous 12 months (75.0%), and those with no diagnosis of dengue in the previous 12 months (68.2%). Bivariate analysis showed no statistical association between these variables and cytomegalovirus seropositivity. Thus, an adjusted analysis was not feasible. 

Approximately 7 out of 10 participants had cytomegalovirus and Epstein**-**Barr virus seropositivity. The seropositivity rates were stratified by sex, age, and ethnicity to assess if there were any differences among the variables (Figure 1). Cytomegalovirus infection was more frequent in non-whites (71.3%), compared with white individuals (57.2%), but no statistical significance was observed among the three variables for cytomegalovirus or Epstein**-**Barr virus seroprevalence. 

**FIGURE 1:**
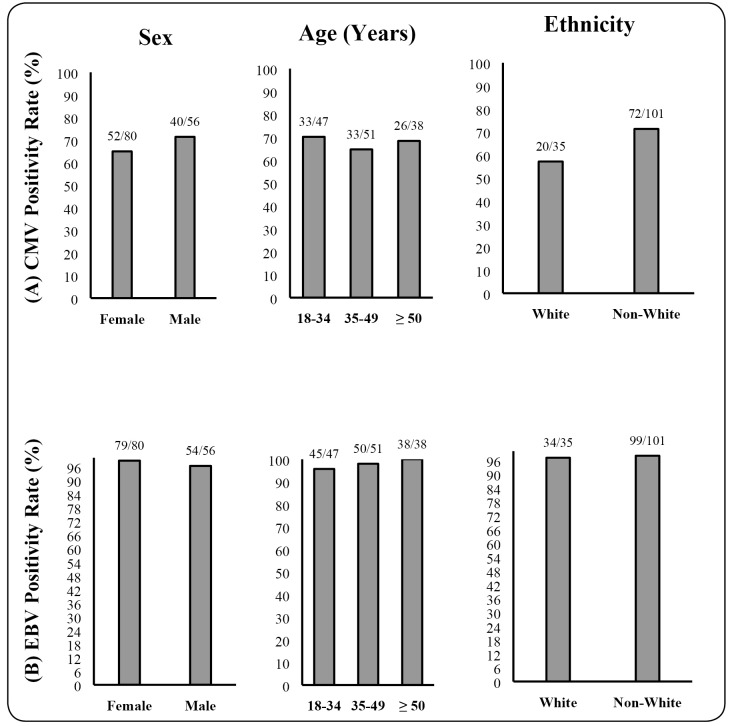
Seropositivity rates (%) stratified by sex, age, and ethnicity for (A) cytomegalovirus infections (CMV) and (B) Epstein**-**Barr virus (EBV) infections in adults in Manaus, 2016.

Our study had limitations, such as those inherent to cross-sectional designs. Although we attempted to contact every participant from the population-based survey, the small sample size of this seroepidemiological study was a weakness. Selection bias may have occurred since the majority of the participants belonged to the lower socioeconomic classifications (C and D/E), probably because of their lower access to health services in comparison to the wealthier population of Manaus^5^. As reported earlier, individuals with lower socioeconomic status are more likely to present with cytomegalovirus seropositivity^6^. It is likely that the sample in our study was not representative of the general population, due convenience sampling. 

As far as we know, this is the first seroepidemiological study conducted in the general population of Manaus. Our results are similar to those of a serological study carried out with 616 Brazilians and 399 Japanese immigrants living in the Northeast region of Brazil in 1989. Cytomegalovirus seropositivity was found in 69.8% of the Brazilian population and 83.7% of the Japanese participants^8^. A cross-sectional study conducted between 1990 and 1991 in Rio de Janeiro showed that 81% of the 121 adult participants admitted to a university hospital were seropositive for cytomegalovirus^9^. Another study carried out in Santa Catarina from 2006 to 2007 found a cytomegalovirus prevalence of 89.3% in 233 solid organ donors^10^. Recently, a cross-sectional study performed with 324 pregnant adolescents between 2009 and 2010 in Belém city showed that IgG seropositivity for cytomegalovirus was found in 96.3% of participants^11^. These differences may be explained by the fact that the participants belonged to specific groups, which may not be representative of the general population.

Our study found no statistical association between the selected independent variables and cytomegalovirus infections. Similar results were reported in a study conducted from 1999 to 2001 in southern Brazil with 115 patients who received liver transplantations. Although the infections were more frequent in men (62.7%), no correlation between age or sex and cytomegalovirus positivity was found^12^. Another study carried out in Salvador city from 2008 to 2010 suggested that there is no significant difference between the sexes with regard to prevalence of cytomegalovirus infections among patients with hematologic disorders. The study reported a significant association in patients aged >58 years^13^.

Despite no significant association, our study suggests that cytomegalovirus seroprevalence is more frequent among non-white individuals. A large study performed in the United States between 1988 and 1994 using cytomegalovirus seroprevalence data from the third National Health and Nutrition Examination Survey (NHANES) investigated the characteristics of 11,859 individuals. The researchers reported that the force of infection was significantly higher in non-Hispanic blacks and Mexican Americans when compared with non-Hispanic whites, indicating that cytomegalovirus is circulating more frequently in non-white individuals^14^. This may be explained in part by factors related to lower socioeconomic status of non-white individuals, as well as the different cultural practices related to breastfeeding, childcare, and sexual activity^2^.

We observed a higher proportion of infections among men when compared with women, but that difference was not significant. This finding differs from previous studies that suggest women are more likely to present cytomegalovirus-IgG seropositivity than men. Higher prevalence in women may be due to greater contact with children, which represents a horizontal mode of transmission to mothers, pregnant women, and those in occupations associated with exposure to children^7^. Our results suggest a higher frequency of cytomegalovirus infections in individuals with health insurance coverage. Data from the third NHANES indicated that individuals who had government-sponsored medical insurance (assisted by public services) were more likely to be seropositive for cytomegalovirus than those with private health insurance, probably because most people with low-coverage health insurance belong to the lower socioeconomic groups^7^.

Seroprevalence of Epstein**-**Barr virus was high, consistent with a previous study conducted from 2016 to 2017, with 578 tissue donors from different regions in Brazil, where the prevalence of IgG antibodies against this virus was 98.3%^15^. The same study reported a cytomegalovirus seroprevalence of 93.0%. Similar to our data, the results of the previous study showed no differences in the prevalence of Epstein**-**Barr virus and cytomegalovirus infections between the sexes or among age groups. Ethnic/racial disparities among participants were not analyzed in the earlier study^15^.

Seroprevalences of cytomegalovirus and its coinfection with Epstein**-**Barr virus were high in Manaus. Due to the underpowered sample, no associations were observed among cytomegalovirus infection and the socio-demographic characteristics.
